# Burden of cancers attributable to modifiable risk factors in Malaysia

**DOI:** 10.1186/s12889-021-10412-9

**Published:** 2021-02-26

**Authors:** H. S. Teh, Y. L. Woon

**Affiliations:** Centre for Clinical Epidemiology, Institute of Clinical Research, National Institute of Health, Ministry of Health Malaysia, Persiaran Setia Murni, Setia Alam, 40170 Shah Alam, Selangor Malaysia

**Keywords:** Population attributable fraction, Modifiable risk factor, Burden, Cancer, Tobacco smoking, Excess weight, Alcohol intake, Physical inactivity, Malaysia

## Abstract

**Background:**

This is a systematic assessment of the burden of cancers in Malaysia in 2018 using epidemiologic approach. The purpose of this study was to identify the proportion of cancers in Malaysia that were attributable to the modifiable risk factors of excess weight, alcohol intake, physical inactivity, tobacco smoking and to estimate the number of cancer cases that could be prevented if the exposure to the modifiable risk factor was reduced.

**Methods:**

We estimated the Population Attributable Fraction (PAF) of the modifiable risk factors to cancers incidences in Malaysia. The two parameters used for the estimation were exposure prevalence from national representative surveys and the relative risk of getting the cancers from worldwide literature review.

**Results:**

Among 38,426 cancer incidences in 2018 from Globocan data, we estimated that 22.2% (95% confidence interval (CI):14.9 to 29.6%) of the cancer incidences included in this study were attributable to the investigated modifiable risk factors. 39.1% (95% CI:27.2 to 49.7%) and 10.5% (95% CI:5.8 to 15.7%) of cancers in male and female respectively, were attributable to the studied modifiable risk factors. The top main cancers attributed by the risk factors were lung cancer (65.1%; 95% CI:56.4 to 72.9%), laryngeal cancer (63.6%; 95% CI:39.9 to 80.5%), and oesophageal cancer (51.5%; 95% CI:39.9 to 62.0%). For each risk factor studied across genders, tobacco smoking contributed the most (14.3%; 95% CI:9.9 to 17.3%), followed by excess weight (7.0%; 95% CI:4.1 to 10.2%), physical inactivity (1.0%; 95% CI:0.4 to 1.7%) and alcohol intake (0.6%; 95% CI:0.2 to 1.0%).

**Conclusion:**

Findings from this study suggests that tobacco smoking and excess weight are the two predominant factors out of the four studied risk factors for cancer cases in Malaysia. Nationwide public health prevention campaigns tailored to these risk factors are recommended. However, the other risk factors such as physical inactivity and alcohol intake shall not be neglected. PAFs are estimated based on the best available data that we have currently. Regular collection of other risk factor exposure prevalence data is vital for future analyses.

**Supplementary Information:**

The online version contains supplementary material available at 10.1186/s12889-021-10412-9.

## Background

The incidence of cancer has been increasing over the years and the battle to reduce this disease burden has been ongoing for decades. In fact, cancer is the second leading cause of death globally and an estimated 9.6 million deaths in 2018 was due to cancer. The World Health Organization (WHO) shared that currently, there are 24.6 million people living with cancer, and by 2020 it is projected that there will be 16 million new cancer cases and 10 million cancer deaths yearly [1]. Unfortunately, from these massive estimates, around 70% of deaths due to cancer occur in low- and middle-income countries.

In Malaysia, numerous efforts have been emphasized in the National Cancer Control Programme to combat this healthcare crisis, which mainly directed at prevention, early detection, improved treatment and palliative care. Prevention approach can be cost-effective and can be introduced at an individual level. According to the World Health Organization (WHO), between 30 and 50% of cancers can currently be prevented by avoiding risk factors and implementing existing evidence-based prevention strategies [1]. In order for prevention approaches to be successful, we have to identify the magnitude of potential risk factors that could attribute to cancer incidences. The cause for cancers can be multifactorial. Some of the risk factors can be avoided by modifying lifestyles and reducing exposures. In regards to this, tobacco use, alcohol use, excess weight, and physical inactivity are recognized as major cancer risk factors worldwide. These are also the shared risk factors for other non-communicable diseases [2].

From the epidemiological point of view, the quantification of the burden of cancer due to risk factors can be estimated by the Population Attributable Fraction (PAF) approach. This concept was first proposed by Levin in 1953, using standard formulae incorporating exposure prevalence to the risk factors and its respective relative risk data [3]. PAF estimate is interpreted as the proportion of cases that could have been prevented if the exposure to risk factors is reduced to the ideal reference level [4–6]

Globally, there are a number of studies that have looked into the burden of cancer using the PAF approach. Most of the studies were done in high-income countries owing to the extensive availability of health expenditure data. Assessment of risk factors attributed to cancers that have been conducted include smoking [7], body mass index [8–10], alcohol [11], physical inactivity [12]. There were a number of papers that look into multiple cancer sites and risk factors, and the magnitudes can differ across countries [13–17].

In Malaysia, potential reductions in colorectal cancer cases attributable to the modifiable risk factors (alcohol, physical inactivity and overweight) have been published in 2017 [18]. From this study, it was highlighted that 18% of colorectal cancer cases in Malaysia could have been prevented through preventive measures. Nevertheless, this study only focused on the incidences of colorectal cancer attributed by individual and combined risk factors of overweight, physical inactivity and alcohol intake. To our knowledge, there are no established studies in Malaysia on the burden of different types of cancer due to different risk factors from epidemiological approach. Therefore, this present study aims to identify the proportion of cancers in Malaysia that are attributable to selected modifiable risk factors, and to estimate the number of cancer cases that could be prevented if the exposure to the modifiable risk factor is reduced.

The values obtained from this study would be particularly useful for public health experts to prioritise the prevention approach at national level. Furthermore, the findings from this study could contribute to the 5-year planning of the Malaysia National Cancer Control Programme in 2021.

## Methods

The methodological framework used in this study was adopted from The Canadian Population Attributable Risk of Cancer project [19]. Under this framework, Population Attributable Fraction (PAF) of the modifiable risk factors to cancers incidences in Malaysia was estimated. The two parameters used for the estimation of PAF were exposure prevalence to selected risk factors and the relative risk (RR) of getting the cancers given the exposure.

### Selection and exposure prevalence of risk factors

The selection of risk factors was determined based on convincing evidence from global wide meta-analysis. The risk factors chosen for this study were overweight and obesity, alcohol intake, physical inactivity and tobacco smoking. The decision for risk factors was largely driven by the availability of prevalence data for Malaysian population. There were other convincing modifiable risk factors (such as dietary and infection) that could not be included in the analysis due to lacking of related exposure prevalence data. There were a few assumptions taken for the selection of risk factors, such as below:

1. The relationship between the risk factors and cancers was either convincing or probable based on the International Agency for Research on Cancer (IARC) and World Cancer Research Fund (WCRF) report [20, 21].
2. Latency period of approximately 12 years was assumed between exposure to risk factors and cancer occurrence.
3. The effects of risk factors on each cancer were independent from each other.

Exposure prevalence was taken from National Health and Morbidity Survey 2006 (NHMS 2006) [22]. This survey was a national household survey conducted once every 10 years to look at health status and risk factors for non-communicable diseases (NCD) in Malaysia. It involved 10,000 living quarters that were randomly selected nationwide and approximately 40,000 respondents were interviewed using standard questionnaire. Individuals were considered overweight if their body mass index (BMI) was between 25 kg/m^2^ and 29.99 kg/m^2^ and obese if their body mass index was 30 kg/m^2^ or greater. Physical inactivity was defined as having a total physical level (occupational plus recreational) of less than 600 metabolic equivalents-minutes per week. Alcohol intake in this study was taken as current drinker, which was defined as those who still consumed alcohol for the past 1 month prior to the NHMS survey. Tobacco smoking prevalence was taken as those who were current smoker and former smoker. The multi-level categorical exposure data available for this analysis were sex-specific but not age-specific.

### Relative risk (RR) to cancers

RRs to selected risk factors (overweight and obesity, physical inactivity, alcohol intake and tobacco smoking) were obtained from literature review. Meta-analyses of cohort studies were preferred, followed by meta-analyses of case-control studies. RRs were selected based on compatibility with the exposure prevalence data and sex-specific RRs were used where available. The references for meta-analysis used for each risk factors were in the additional file. The reference category used in the RR sources of alcohol intake and tobacco smoking was ‘unexposed’ to the risk factors. For physical activity, exposure prevalence was calculated as deficit against the reference category in the RR source (600 metabolic equivalent [MET]-minutes per week). For body mass index, the reference category used was the normal weight (< 25 kg/m^2^).

RR was provided for the presence of physical activity, therefore, conversion of relative risk due to physical inactivity was calculated as the natural logarithm of the reciprocal of the RR (ln(1/RR)) [23].

### Cancer incidence data

Cancer incidences in Malaysia for year 2018 were obtained from Global Cancer Observatory (Globocan) analysis [24]. This platform provided most recent data in relation to cancer incidences in Malaysia in 2018. The incidence data used were not based on subtypes or morphological types. A total of 21 types of cancer were included in the analysis of this current study, based on its established causal relationship to the risk factors selected and also the availability of incidence data. Those cancers were oesophageal, pancreas, liver, colorectal, breast, endometrial, kidney, oral cavity, nasopharynx, larynx, stomach, gallbladder, bladder, ovary, prostate, cervix, lung, Non-Hodgkin’s lymphoma, Hodgkin’s lymphoma, leukaemia and multiple myeloma.

### Calculation of PAF

Individual PAFs were calculated for each risk factor for each cancer type based on Eq. 1. Overweight and obesity were regarded as a trichotomous exposure level to excess body weight as 3 categories of exposure were involved: normal body weight, overweight and obesity. Tobacco smoking was also regarded as a trichotomous exposure level based on 3 categories of exposure involved: non-smoker, current smoker and former smoker. Combined PAFs were then calculated using Eq. 2 for each cancer type. In this study, the maximum risk factors included in the analysis can be four types (excess weight, alcohol intake, physical inactivity and tobacco smoking). Where relevance for the cancer types based on causal evidences from WCRF, less than four risks factors can be included for analysis.

### PAF estimation based on a single risk factor

Individual PAF due to single risk factor was calculated by the following formulae [3, 13].

1 $$ \mathrm{Individual}\ \mathrm{PAF}=\frac{\left[{\mathrm{P}}_1\left({\mathrm{ERR}}_1\right)+{\mathrm{P}}_2\left({\mathrm{ERR}}_2\right)..+{\mathrm{P}}_{\mathrm{n}}\left({\mathrm{ERR}}_{\mathrm{n}}\right)\right]}{\left[{\mathrm{P}}_1\left({\mathrm{ERR}}_1\right)+{\mathrm{P}}_2\left({\mathrm{ERR}}_2\right)..+{\mathrm{P}}_{\mathrm{n}}\left({\mathrm{ERR}}_{\mathrm{n}}\right)\right]+1} $$

Where, P = Prevalence of exposure level 1 (and so on) in the population.

ERR = Excess relative risk at exposure level 1 (and so on), ERR = RR-1.

### PAF estimation based on multiple risk factors

In this present study, we calculated PAFs for all risk factors combined using eq. 2. This aggregation formulae did not account for the synergistic effect of different risk factors on the cancer risk.

2 $$ {\mathrm{PAF}}_{\mathrm{c}\mathrm{ombined}}=1\hbox{-} \left(1\hbox{-} {\mathrm{PAF}}_{\mathrm{A}}\right)\times \left(1\hbox{-} {\mathrm{PAF}}_{\mathrm{b}}\right)\times \left(1\hbox{-} {\mathrm{PAF}}_{\mathrm{c}}\right)\times \left(1\hbox{-} {\mathrm{PAF}}_{\mathrm{d}}\right) $$

### Estimation of Cancer incidences attributable to modifiable risk factors

We estimated the number of cancer cases attributable to the modifiable risk factor(s) by applying the calculated combined PAFs to the cancer incidences obtained from Globocan 2018. Overall PAF for all cancers was determined by summing the calculated preventable incidences of each cancers and dividing by all cancer incidences investigated in this study for the year 2018. Sensitivity analyses were conducted using the upper and lower confidence intervals of the exposure prevalence data and RRs to calculate the highest- and lowest-possible PAFs.

## Results

The exposure prevalence data extracted from National Health and Morbidity Survey 2006 (NHMS 2006) was summarized in Table S1, Additional File. Sex-specific cancer incidences extracted from Globocan 2018 were summarized in Table S2, Additional File. The relative risk data extracted from meta-analysis was summarized in Table S3, Additional File.

**Fig. 1:**
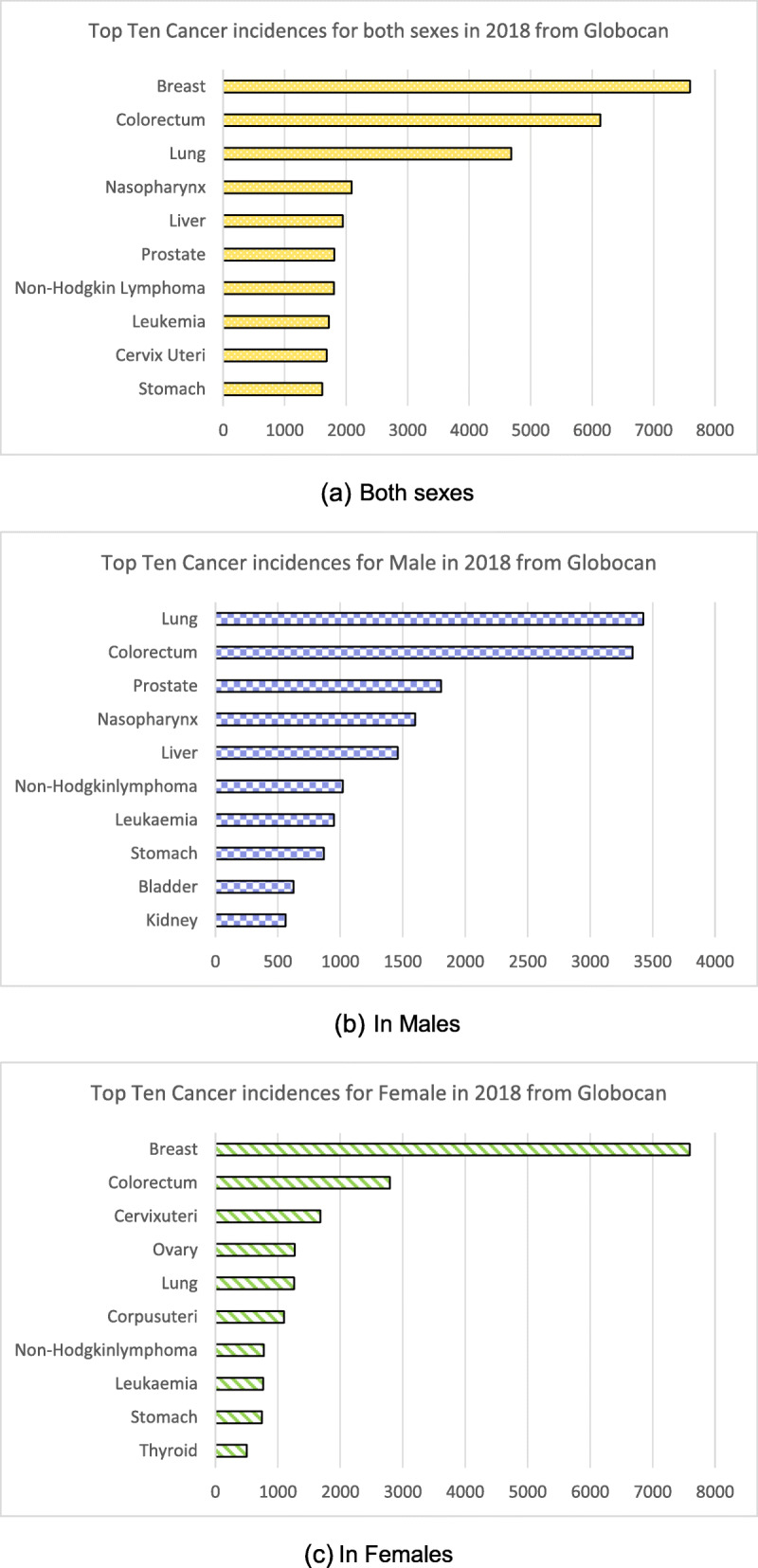
Estimated number of incident cases in Malaysia in 2018 from Globocan. (a) Both sexes, (b) in males and (c) in females. Source:IARC Globocan 2018(https://gco.iarc.fr/)

Based on Globocan data in 2018, the top 10 cancers among Malaysia population were breast, colorectal, lung, nasopharynx, liver, prostate, non-Hodgkin lymphoma, leukaemia, cervix uteri and stomach (Fig. 1).

**Fig. 2:**
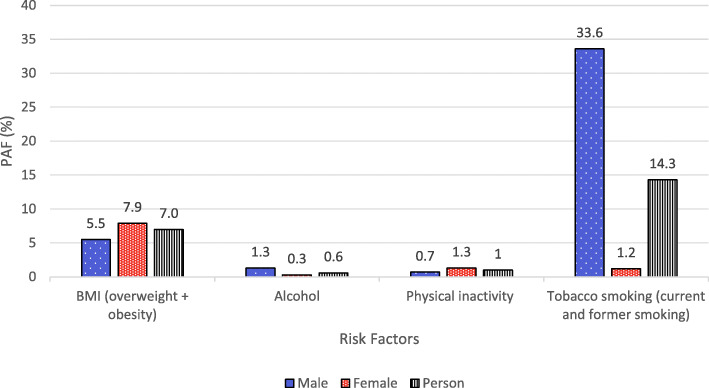
Summary of PAF by types of modifiable risk factors in male, female and persons

Approximately one in four (22.2%; 95% CI:14.9 to 29.6%) cancer incidences included in this study were attributable to the four modifiable risk factors. Looking at risk factors across both sexes, 14.3% (95% CI: 9.9 to 17.3%) of the incidence among the selected cancer types were attributed by smoking (Fig. 2). For the other three factors, the overall PAFs were below 10 %. In general, males have higher PAF for all risk factors than females. Among the four risk factors (Table 1), tobacco smoking contributed the largest proportion of attributable cases (33.6% in male and 1.2% in female). Interestingly, excess weight was a more predominant risk factor for cancer incidences in female. A total of 1652 cancer incidences could have been avoided among females compared to 979 avoidable cases in males if body mass index was reduced. PAF for physical inactivity was also higher in female (1.3%; 95% CI:0.4 to 2.2%) than in male (0.7%; 95% CI:0.3 to 1.0%). For the alcohol intake, PAFs for both sexes were generally low, which were less than 2 %.

**Table 1:** Individual PAFs attributable to excess weight, alcohol intake, tobacco smoking and physical inactivity in Malaysia in 2018

Exposure Category	Attribution to Cancer Incidence in 2018					
	Males		Females		Persons	
	PAF, % (95% CI)	Number, N (95% CI)	PAF, % (95% CI)	Number, N (95% CI)	PAF, % (95% CI)	Number, N (95% CI)
BMI (over weight and obesity)	5.5(2.7–8.8)	979 (472–1561)	7.9 (4.8–11.3)	1652 (990–2349)	7.0 (4.1–10.2)	2684 (1568–3905)
Alcohol intake	1.3 (0.5–2.4)	225 (78–413)	0.3 (0.1–0.5)	53 (19–108)	0.6 (0.2–1.0)	219 (85–386)
Physical Inactivity	0.7 (0.3–1.1)	116 (56–183)	1.3 (0.4–2.2)	263 (75–459)	1.0 (0.4–1.7)	385 (136–654)
Smoking (current and former smoking)	33.6 (22.9–39.7)	5933 (4047–7014)	1.2 (0.6–2.0)	252 (121–422)	14.3(9.9–17.3)	5485 (3812–6654)

Summary results for individual PAF for each risk factors based on cancer sites and its preventable cases were reported in Table S4 and S5 in Additional File. It was worth noting that PAF due to smoking varied greatly, ranging from the least magnitude of 0.9% (ovary cancer) to the largest magnitude of 65.1% (lung cancer). The variation reflected a difference in the relative risk across cancers.

**Fig. 3:**
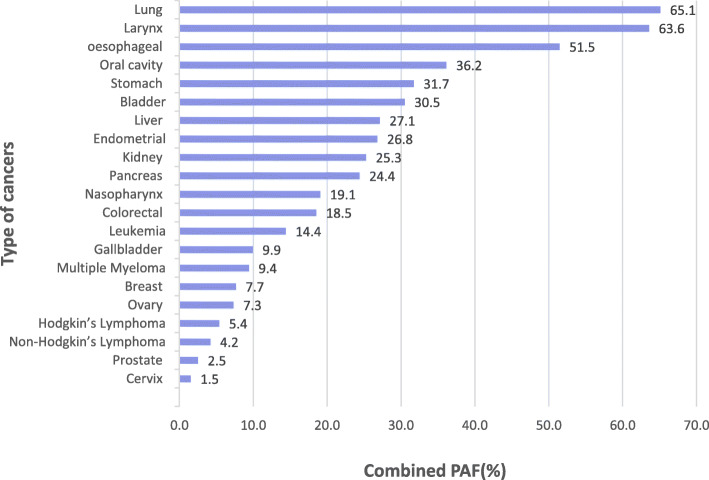
Visual representation of combined PAF (%) by types of Cancer in 2018

Combined PAFs were also different across sexes for all cancers studied here (Table S6 Additional File). Higher combined PAF in males (39.1%; 95% CI: 27.2 to 49.7%) than in females (10.5%; 95% CI:5.8 to 15.7%). Cancers associated with tobacco smoking showed relatively larger PAFs. The cancers that were greatly attributed by all-joint modifiable risk factors were depicted in Fig. 3: lung (65.1%; 95% CI:56.4 to 72.9%), larynx (63.6%; 95% CI:39.9 to 80.5%), oesophageal (51.5%; 95% CI:39.9 to 62.0%), oral cavity (36.2%; 95% CI:22.4 to 49.6%), stomach (31.7%; 95% CI:18.4 to 44.2%), bladder (30.5%; 95% CI:23.3 to 37.8%), liver (27.1%; 95% CI:15.2 to 38.8%), endometrial (26.8%; 95% CI:21.1 to 31.8%), kidney (25.3%; 95% CI:20.0 to 31.0%), pancreas (24.4%; 95% CI:14.1 to 34.8%), nasopharynx (19.1%; 95% CI:6.5 to 32.9%), colorectal (18.5%; 95% CI:10.0 to 27.6%), leukaemia (14.4%; 95% CI:6.3 to 23.2%) and the others (less than 10%). This analysis observed Malaysia had a lower overall PAF than other countries as we only included four modifiable risk factors in the study. The comparison with other countries was summarized in Table 2.

**Table 2:** Comparison of PAF across countries based on similar PAF approach in both sexes

Risk Factors	Malaysia, current study	France, 2000 [25]	China, 2005 [26]	Japan, 2005 [15]	Canada,2013 [27]	Australia, 2013 [16]	UK, 2015 [13, 28]	Vietnam, 2018 [29]
Excess weight	7.0%	1.6%	0.32%	1.1%	4.6%	4.3%	6.3%	0.8%
Alcohol intake	0.6%	6.9%	4.4%	6.3%	3.5%	2.8%	3.3%	6.0%
Physical Inactivity	1.0%	1.6%	0.27%	0.4%	4.1%	1.5%	0.5%	–
Tobacco smoking	14.3%	23.9%	22.6%	19.5%	17.9%	13.3%	15.1%	13.5%
Overall PAF (include all other risk factors)	22.2%	35.0%^a^	57%^b^	42.7%^c^	27.7%	32.8%^d^	37.7^e^	47.0%^f^

^a^Other PAFs include: Infectious agents (3.6%), occupation (2.4%), exogenous hormones (0.9%), ultraviolet light (0.7%), pollutants (0.2%)

^b^Other PAFs include: Infectious agents (29.4%), low fruit intake (13.0%), low vegetable intake (3.6%), occupational agents (2.7%), environmental agents (0.68%), reproductive factors (0.17%) and hormone replacement therapy (0.01%)

^c^Other PAFs include: Passive smoking (0.6%), infectious agents (20.6%), salt intake (1.6%), fruit intake (0.7%), vegetable intake (0.6%), exogenous hormones used (0.2%)

^d^Other PAFs include: Dietary factors (5.4%), infections (3.3%), solar UV radiation (6.4%), reproductive factors (0.8%)

^e^Other PAFs include: UV radiation (3.8%), occupation (3.8%), infections (3.6%), insufficient fibre (3.3%), ionising radiation (1.9%), processed meat (1.5%), air pollution (1.0%), not breastfeeding (0.5%), postmenopausal hormones (0.4%), oral contraceptives (0.2%)

^f^Other PAFs include: Passive smoking (6.0%), infectious agents (29.1%), low vegetable and fruit intake (0.4%), air pollution (1.5%), nulliparity (0.8%)

## Discussion

This is the first study in Malaysia that summarized the burden of cancer attributed to selected modifiable risk factors. Our study estimated that approximately one-fourth of overall cancers studied in this paper were potentially preventable by adjusting one of the four risk factors (excess weight, alcohol intake, physical inactivity and smoking).

### Variation by cancer types and gender

Out of the 21 types of cancers studied in this paper, only colorectal cancer was attributed by joint effects of all 4 types of modifiable risk factors (Table S2). Although breast cancers and colorectal cancers were the top cancers affecting Malaysian population, the estimated PAFs were lower (less than 20%). Notably, PAFs estimates would be higher if the risk factors were either those with high exposure prevalence among the population, those with strong causal relationship with the associated cancers (high relative risks) or a combination of both. From the paper by Krueger et al. [30] that studied the same four risk factors for 2013 cancer incidences in Canada, the combined PAF for breast cancers and colorectal cancers were 21.8 and 50.6% respectively. This very much higher PAF reflected variation in exposure prevalence and relative risk to the risk factors among Canadian [27]. For studies conducted among Asian population, combined PAFs were higher for colorectal and breast cancer due to incorporation of more risk factors in the analysis, such as dietary intake, exogenous hormone use, reproductive factor, occupational and environmental agents. The combined PAFs ranged from 10.5–26.1% for breast cancer and 8.7–33.6% for colorectal cancers [15, 26, 29]. This suggested that further studies related to more additional risk factors can be conducted to investigate the underlying modifiable risk factors in Malaysia.

Gender-specific social behaviours such as tobacco smoking and alcohol intake might explain the difference in overall PAF by sexes. This trend was similar in any other countries in the United Kingdom, where men generally had higher relative risks and also exposure prevalence [13]. In China, discrepancies in cancer-specific PAF between male (combined PAF = 65.9%) and female (combined PAF = 42.8%) were also explained by the difference frequency of exposure to important risk factors, such as smoking, alcohol drinking and chronic infection [26].

### Variation by risk factors

Out of the 4 modifiable risk factors chosen for the study, more of the included cancers were associated with excess weight and smoking, compared to alcohol intake and physical inactivity. Specifically, physical inactivity only increased the risk of two types of cancers in this study (colorectal and breast). Looking at cancer types, this study showed that lung cancer, laryngeal cancer and oesophageal cancer had the highest proportion of potentially avoidable cases. The risk factor associated with these top three estimates was largely by tobacco smoking. This finding was consistent with the fractions of cancer reported by a global review done in 2016, in which more than half of the lung and laryngeal cancer were attributed by smoking [4].

Consistently, tobacco smoking was identified as the major risk factor across the continents, with magnitudes range from 13.3 to 23.9% (13,15,16,26– [25]. From our study, tobacco smoking contributed largely with 14.7% reported PAF for all cancers studied here. This figure was half of the burden of cancer PAF estimate done in Association of Southeast Asian Nations (ASEAN) countries in 2016, with a reported average value of 28.4% [7]. The difference in the PAF values was due to the selection of the denominator used in the PAF calculation. The ASEAN study only looked into the effect of tobacco smoking alone, therefore, the PAF was calculated using smoking attributable cancer incidences. Our study looked into multiple risk factors on burden of cancer and all 21 cancer incidences were taken into calculation. The high estimate of our country PAF on tobacco smoking was mainly contributed by the high prevalence among males’ population (46.4% prevalence in men and 1.6% in women). In other countries, prevalence of tobacco smoking was relatively similar among males and females, such as in Canada (18.8% in males and 13.4% in females) and in United Kingdom (22% in males and 19% in females) [27, 28]. In comparison to Southeast Asia country such as Vietnam, the reported study done by Nguyen et al.^27^ did not include a few cancers for which a causal association with tobacco smoking has been demonstrated, such as colorectal and kidney cancers [29]. Therefore, our PAF estimate was higher even though the exposure prevalence in Vietnam outnumbered us (60.1% in males and 1.9% in females). In addition to active smoking, literature showed that passive smoking also played as important factor for cancer development, an analysis in Vietnam which included these factors showed a PAF of 8.8% among women. Our finding could be an underestimation of true effect causing by smoking as we did not take into account of passive smoking as a risk factor due to unavailability of prevalence data for passive smoker [29].

The second leading risk factor for cancers in Malaysia was excess weight. Alarmingly, Malaysia has similar PAF (7.2%) to western countries such as in Australia (4.3%), Canada (4.6%) and United Kingdom (6.3%) [13, 16, 27]. The value was far different from Asian countries like China, Japan and Vietnam with PAF of 0.32–1.1% [15, 26, 29]. The possible reason could be due to the high prevalence of excess weight/obesity among Malaysian population. The prevalence of overweight and obesity among adults (18 years and above) has increased tremendously over the years, from 16.6 and 4.4% respectively in 1996 to 30.0 and 17.7% respectively in 2015 [31, 32]. Malaysia is also first in the rank for overweight and obesity among South East Asia [33]. Furthermore, the latest NHMS 2019 showed that one in two people in Malaysia was overweight/obese [34]. This increment signified that the number of cancer attributable to overweight would be much higher in the next 10 years or so if no intervention is implemented, considering this study looked upon the exposure prevalence in 2006 for the cancer incidences in 2018. Alarmingly, these risk factors also interlinked to other non-communicable diseases such as diabetes, heart disease and hypercholesterolemia.

Physical inactivity appeared to be the third leading factor for all cancers, with an overall PAF of 1.0%. Although the effect of physical inactivity was only on two types of cancers, its attribution should not be ignored. Again, our physical inactivity PAF value was comparable to western countries than to Asian countries listed in Table 2. This showed that Malaysian population seemed to have a sedentary lifestyle similar to developed countries.

Based on our analysis, alcohol intake had the least attribution to overall cancer, which was 0.6%. This was much lower than the values of other countries listed in Table 2. This discrepancy could be due to cultural factors, in which alcohol intake prevalence was relatively low in Malaysia (prevalence 13.7% in men and 0.4% in women). In Malaysia, majority of the population are Muslim (60%), in which alcohol intake is prohibited in Islamic teaching. From the study in Vietnam, a remarkable high prevalence of alcohol intake among men was noticed in Vietnam (82%), in China (39%) and globally (55%) [29]. Also, the exposure prevalence category for alcohol intake was only limited to current drinker in this study due to unavailability of prevalence data based on dose-response relationship. The other papers commonly quoted alcohol drinker as light, medium and heavy drinker, which accounted for higher magnitudes of PAFs.

### Strength and limitations

Overall, this study had a number of strengths. First and foremost, this was the first study in Malaysia that compiled PAF based on the most common risk factors in the local population. Also, the calculation involved the exposure prevalence from large scale representative data in the local setting. Secondly, it utilized the most recent relative risk for cancers from available meta-analysis due to common risk factors of excess weight, alcohol intake, physical inactivity and smoking.

Nevertheless, we acknowledged that our study had a few limitations. The estimated 12-year latency period used for the cancer outcome has always been debatable. For risk factors like tobacco smoking, the latency period from exposure to cancer development could have been longer. Due to restriction by the exposure prevalence data availability, we could only retrieve the Malaysian population data pertaining to selected risk factors in 2006. Secondly, the relative risk estimates obtained from meta-analysis were not specific to the population in Malaysia. Due to lacking of cohort studies done in Malaysia pertaining to the risk factors, meta-analysis could not be conducted. Also, the inclusion of risk factors in PAF assessment was largely driven by the availability of the data pertaining to the cancers. We acknowledged there were other causal factors such as diet, pollution, genetic inheritance and infections that could have contributed to incidence of cancers. Therefore, for this study, we can only analyse the 21 types of cancers which were shown to have established causal relationships with the risk factors than choosing all cancers.

In terms of PAF calculation, overall unadjusted RRs were mainly used in this analysis and sex-specific RRs were used to a lesser extent. The assumption of no other confounding factors (e.g. age) for the relative risks used may cause the estimates to be biased and the magnitude of bias could be related to the strength of confounding [35]. The unavailability of incidence data for cancer subtypes and morphological types such as oesophageal squamous cell carcinoma, pre- and post-menopausal breast cancer, gastric cardia and advanced prostate cancer could have overestimated the calculated PAFs. Estimates from Globocan for Malaysia’s cancer incidence was used in the analysis. The accuracy of its estimation had great influence towards the reliability of our analysis. Nevertheless, the estimates from Globocan provides us with more recent country cancer incidence than the published country report.

Furthermore, Eq. 3 assumed exposures to the risk factors were statistically independent, whereby individual experienced one risk factor would be less likely to experience the others. In the real world, interactions between risk factor does occurred and the assumption was made to simplify the quantitative measurement. For any future studies, weighted-sum method to calculate PAFs should be explored to gain more accurate results. From this study, the combined PAFs may not be comprehensive as only four risk factors were included for analysis, however, it provided the minimum benefits that we could gain if the potential exposure to the studied risk factors was reduced.

## Conclusion

As a conclusion, approximately one-fourth of the cancers investigated in this study can be prevented by adjusting the modifiable risk factors. Although it can be an estimate, this systematic assessment managed to pull together the different risk factors into one digestible figure for policy makers to deduce the practical way for cancer prevention in Malaysia. Future studies should incorporate other risk factors such as physical carcinogens (ionising radiation) and biological carcinogens (infections) to get a better understanding of the magnitude of each risk factor to respective cancer. From this study, it has re-emphasized that tobacco smoking and excess weight are the two predominant factors out of the four studied risk factors for cancer cases in Malaysia. Continuous effort to introduce and sustain prevention programmes related to these two risk factors is very much needed. In the future, regular collection of other risk factor exposure prevalence data is important for such analyses.

## Supplementary Information

**Additional file 1.**

## Data Availability

The datasets used and/or analysed during the current study were listed in the reference list of the Additional File. Those data were publicly available and no permissions were required in order to access the data.

## Abbreviations

ASEAN: Association of Southeast Asian Nations
BMI: Body mass index
CUP: Continuous update project
GLOBOCAN: Global cancer observatory
IARC: International agency for research on cancer
MET: Metabolic equivalents
NCD: Non-communicable diseases
NHMS: National health and morbidity survey
PAF: Population attributable fraction
WCRF: World cancer research fund report
WHO: World health Organization

## References

[1] (WHO) WHO. Cancer Fact Sheets [Internet]. 2018 [cited 2020 May 4]. Available from: https://www.who.int/news-room/fact-sheets/detail/cancer

[2] World Health Organization (WHO). Western Pacific Regional Action Plan For Noncommunicable Diseases. 2009.

[3] Levin ML (1953). The occurrence of lung cancer in man. Acta Unio Int Contra Cancrum.

[4] Whiteman DC, Wilson LF. The fractions of cancer attributable to modifiable factors: a global review. Cancer Epidemiol 2016;44:203–221. Available from: http://dx.doi.org/10.1016/j.canep.2016.06.013 10.1016/j.canep.2016.06.013 27460784

[5] Whiteman DC, Webb PM, Green AC, Neale RE, Fritschi L, Bain CJ (2015). Cancers in Australia in 2010 attributable to modifiable factors: summary and conclusions. Aust N Z J Public Health.

[6] Shield KD, Parkin DM, Whiteman DC, Rehm J, Viallon V, Micallef CM, Vineis P, Rushton L, Bray F, Soerjomataram I (2016). Population attributable and preventable fractions: Cancer risk factor surveillance, and Cancer policy projection. Curr Epidemiol Rep.

[7] Kristina SA, Endarti D, Thavorncharoensap M. Burden of cancer attributable to tobacco smoking in member countries of the Association of Southeast Asian Nations (ASEAN), 2012. Cancer Epidemiol 2016;44:84–90. Available from: http://dx.doi.org/10.1016/j.canep.2016.08.005 10.1016/j.canep.2016.08.005 27513722

[8] Arnold M, Pandeya N, Byrnes G, Renehan AG, Stevens GA, Ezzati M, Jacques Ferlay, Miranda JJ, Romieu I, Dikshit R, Forman D (2015). Global burden of cancer attributable to high body-mass index in 2012: a population-based study. Lancet Oncol.

[9] Parr CL, Batty GD, Lam TH, Barzi F, Fang X, Ho SC (2010). Body-mass index and cancer mortality in the Asia-Pacific cohort studies collaboration: pooled analyses of 424 519 participants. Lancet Oncol.

[10] Renehan AG, Tyson M, Egger M, Heller RF, Zwahlen M (2008). Body-mass index and incidence of cancer: a systematic review and meta-analysis of prospective observational studies. Lancet..

[11] Fedirko V, Tramacere I, Bagnardi V, Rota M, Scotti L, Islami F (2011). Alcohol drinking and colorectal cancer risk: an overall and dose-response meta-analysis of published studies. Ann Oncol.

[12] Ding D, Lawson KD, Kolbe-Alexander TL, Finkelstein EA, Katzmarzyk PT, van Mechelen W, et al. The economic burden of physical inactivity: a global analysis of major non-communicable diseases. Lancet 2016;388(10051):1311–1324. Available from: http://dx.doi.org/10.1016/S0140-6736(16)30383-X 10.1016/S0140-6736(16)30383-X 27475266

[13] Brown KF, Rumgay H, Dunlop C, Ryan M, Quartly F, Cox A, et al. The fraction of cancer attributable to modifiable risk factors in England, Wales, Scotland, Northern Ireland, and the United Kingdom in 2015. Br J Cancer. 2018;118(8):1130-41. 10.1038/s41416-018-0029-6 PMC5931106 29567982

[14] Charafeddine MA, Olson SH, Mukherji D, Temraz SN, Abou-Alfa GK, Shamseddine AI (2017). Proportion of cancer in a middle eastern country attributable to established risk factors. BMC Cancer.

[15] Inoue M, Sawada N, Matsuda T, Iwasaki M, Sasazuki S, Shimazu T, et al. Attributable causes of cancer in Japan in 2005-systematic assessment to estimate current burden of cancer attributable to known preventable risk factors in Japan. Ann Oncol 2012;23(5):1362–1369. Available from: http://dx.doi.org/10.1093/annonc/mdr437 10.1093/annonc/mdr437 22048150

[16] Wilson LF, Antonsson A, Green AC, Jordan SJ, Kendall BJ, Nagle CM (2018). How many cancer cases and deaths are potentially preventable? Estimates for Australia in 2013. Int J Cancer.

[17] Islami F, Goding Sauer A, Miller KD, Siegel RL, Fedewa SA, Jacobs EJ (2018). Proportion and number of cancer cases and deaths attributable to potentially modifiable risk factors in the United States. CA Cancer J Clin.

[18] Naing C, Lai PK, Mak JW (2017). Immediately modifiable risk factors attributable to colorectal cancer in Malaysia. BMC Public Health.

[19] Brenner DR, Poirier AE, Walter SD, King WD, Franco EL, Demers PA, et al. Estimating the current and future cancer burden in Canada: Methodological framework of the Canadian population attributable risk of cancer (ComPARe) study. BMJ Open. 2018;8(7):e022378. 10.1136/bmjopen-2018-022378 PMC6074628 30068623

[20] World Cancer Research Fund/American Institute for Cancer Research. Diet, Nutrition, Physical Activity and Cancer: A Global Perspective. Continuous Update Project Expert Report 2018. [Internet]. Available from: dietandcancerreport.org

[21] IARC Working Group on the Evaluation of Carcinogenic Risks to Humans. Personal habits and indoor combustions. Volume 100 E. A review of human carcinogens. IARC Monogr Eval Carcinog Risks Hum. 2012;100(Pt E):1–538. PMC4781577 23193840

[22] Institute for Public Health (IPH). The Third National Health and Morbidity Survey 2006 (NHMS III). 2008.

[23] Olsen CM, Wilson LF, Nagle CM, Kendall BJ, Bain CJ, Pandeya N (2015). Cancers in Australia in 2010 attributable to insufficient physical activity. Aust N Z J Public Health.

[24] Global Cancer Observatory: Cancer Today. Lyon: International Agency for Research on Cancer. https://gco.iarc.fr/today. Accessed 4 May 2020.

[25] Boffetta P, Tubiana M, Hill C, Boniol M, Aurengo A, Masse R (2009). The causes of cancer in France. Ann Oncol.

[26] Wang JB, Jiang Y, Liang H, Li P, Xiao HJ, Ji J (2012). Attributable causes of cancer in China. Ann Oncol.

[27] Krueger H, Andres EN, Koot JM, Reilly BD (2016). The economic burden of cancers attributable to tobacco smoking, excess weight, alcohol use, and physical inactivity in Canada. Curr Oncol.

[28] Parkin DM. The fraction of cancer attributable to lifestyle and environmental factors in the UK in 2010: introduction. Br J Cancer 2011;105(S2):S2–S5. Available from: http://dx.doi.org/10.1038/bjc.2011.474 10.1038/bjc.2011.474 PMC3252063 22158314

[29] Nguyen TP, Luu HN, Nguyen MVT, Tran MT, Van Tuong TT, Du Tran CT, et al. Attributable causes of Cancer in Vietnam. JCO Glob Oncol. 2020;6:195–204. 10.1200/JGO.19.00239 PMC7051248 32045545

[30] National Cancer Registry. Malaysian national cancer registry report 2012–2016: Ministry of Health Malaysia; 2019. https://www.moh.gov.my/moh/resources/Penerbitan/Laporan/Umum/2012-2016%20(MNCRR)/MNCR_2012-2016_FINAL_(PUBLISHED_2019).pdf. Accessed 27 May 2020.

[31] Institute for Public Health (IPH). National Health and Morbidity Survey 2015 (NHMS 2015). Vol. II: Non-Communicable Diseases, Risk Factors & Other Health Problems. 2015.

[32] Institute for Public Health (IPH). National Health and Morbidity Study 1996 (NHMS 2). 1996.

[33] World Health Organization. Malaysia and WHO call for more investment in primary health care the 21st century. https://www.who.int/malaysia/news/detail/08-04-2019-malaysia-and-who-call-for-more-investment-in-primary-health-care-the-21st-century. Accessed 27 May 2020.

[34] National Health and Morbidity Survey 2019: Non-communicable diseases, healthcare demand and health literac [Internet]. 2019. Available from: http://www.iku.gov.my/nhms/

[35] Steenland K, Armstrong B (2006). An overview of methods for calculating the burden of disease due to specific risk factors. Epidemiology..

