# Supplementary material for: Burden of cancers attributable to modifiable risk factors in Malaysia
Source: BMC Public Health. 2021 Feb 26;21:410. doi: 10.1186/s12889-021-10412-9 (PMC7908668; doi:10.1186/s12889-021-10412-9)
Supplement: Supplementary file 1 — Additional file 1. [file 12889_2021_10412_MOESM1_ESM.docx]

**Additional File**

**Table S1** Exposure prevalence data extracted from National Health and Morbidity Survey 2006 (NHMS 2006)[1]

| **Risk Factors** | **Prevalence (%) (95% CI)** | | |
| --- | --- | --- | --- |
|  | **Male** | **Female** | **Persons** |
| BMI | | | |
| Overweight (BMI 25<30) | 29.7 (28.9-30.5) | 28.6 (27.9-29.3) | 29.1 (28.6-29.7) |
| Obesity (BMI ≥30) | 10 (9.5-10.5) | 17.4 (16.7-18.0) | 14(13.6-14.5) |
| Alcohol Intake (current drinker) | 13.7 (12.6-14.7) | 4.0 (3.5-4.5) | 8.5 (7.8-9.1) |
| Physical Inactivity (total MET minutes per week less than 600minutes) | 35.3 (34.3-36.3) | 50.5 (49.5-51.5) | 43.7 (42.9-44.5) |
| Cigarette smoking | | | |
| Current smoker | 46.4 (45.5-47.4) | 1.6 (1.4-1.8) | 21.5 (21.0-22.0) |
| Former smoker | 11.0 (10.4-11.5) | 0.9 (0.7-1.0) | 5.4 (5.1-5.6) |

**Table S2** Cancer incidences 2018 from GLOBOCAN and related attributing modifiable risk factors[2]

| **Types of Cancer** | **ICD 10 Code** | **Attributing Risk Factors** | **Incidences**  **In Male** | **Incidences in Female** |
| --- | --- | --- | --- | --- |
| Oesophageal | C15 | Overweight and obesity, Smoking | 298 | 124 |
| Pancreas | C25 | Overweight and obesity, Smoking | 539 | 437 |
| Liver | C22 | Overweight and obesity, Alcohol, Smoking | 1460 | 484 |
| Colorectal | C18-C21 | Overweight and obesity, Alcohol, Physical Inactivity, Smoking | 3342 | 2795 |
| Breast | C50 | Overweight and obesity, Alcohol, Physical Inactivity | NA | 7593 |
| Endometrial | C54 | Overweight and obesity | NA | 1100 |
| Kidney | C64 | Overweight and obesity, Smoking | 564 | 323 |
| Oral cavity | C00-06 | Alcohol, Smoking | 335 | 332 |
| Nasopharynx | C11 | Alcohol, Smoking | 1600 | 489 |
| Larynx | C32 | Alcohol, Smoking | 432 | 38 |
| Stomach | C16 | Overweight and obesity, Alcohol, Smoking | 868 | 745 |
| Gallbladder | C23-24 | Overweight and obesity | 148 | 201 |
| Bladder | C67 | Smoking | 625 | 194 |
| Ovary | C56 | Overweight and obesity, Smoking | NA | 1271 |
| Prostate | C61 | Overweight and obesity | 1807 | NA |
| Cervix | C53 | Smoking | 0 | 1682 |
| Lung | C34 | Smoking | 3426 | 1260 |
| Non-Hodgkin’s Lymphoma | C82-86, C96 | Overweight and obesity | 1022 | 777 |
| Hodgkin’s Lymphoma | C81 | Overweight and obesity | 134 | 73 |
| Leukaemia | C91-95 | Overweight and obesity, Smoking | 949 | 768 |
| Multiple Myeloma | C88,C90 | Overweight and obesity | 127 | 100 |
| **Total by sexes** | | | **17676** | **20786** |
| **Total incidences 2018** | | | **38462** | |

**Table S3** Relative risk data extracted from meta-analysis

| **Risk Factor^a^** | **Cancer Type** | | | | | | | | | | | | | | | | | | | | |
| --- | --- | --- | --- | --- | --- | --- | --- | --- | --- | --- | --- | --- | --- | --- | --- | --- | --- | --- | --- | --- | --- |
|  | **Oesophageal** | **Pancreas** | **Liver** | **Colorectal** | **Breast** | **Kidney** | **Oral** | **Nasopharynx** | **Larynx** | **Stomach** | **Gallbladder** | **Bladder** | **Ovary** | **Endometrial** | **Prostate** | **Cervix** | **Lung** | **Non-Hodgkin’s Lymphoma** | **Hodgkin’s Lymphoma** | **Leukaemia** | **Multiple Myeloma** |
| **BMI** [3–21] | | | | | | | | | | | | | | | | | | | | | |
| ***Overweight vs healthy weight (BMI 25<30 vs BMI 18.5<25)*** | | | | | | | | | | | | | | | | | | | | | |
| Male | 1.87 (1.61-2.17) | 1.25 (1.10-1.42) | 1.18 (1.06-1.31) | 1.21 (1.07-1.38) | NA | 1.22 (1.09-1.36) | LR | LR | LR | 1.40 (1.18-1.66) | 1.01 (1.00-1.12) | LR | NA | NA | 1.04 (1.00-1.15) | NA | LR | 1.06 (1.00-1.12) | LR | 1.05 (1.01-1.13) | 1.12  (1.06-1.19) |
| Female | 1.87 (1.61-2.17) | 1.25 (1.10-1.42) | 1.18 (1.06-1.31) | 1.21 (1.07-1.38) | 1.10 (1.06-1.13) | 1.48 (1.31-1.67) | LR | LR | LR | 1.40 (1.18-1.66) | 1.22 (1.09-1.37) | LR | 1.08 (1.00-1.31) | 1.34  (1.20-1.48) | NA | LR | LR | 1.06 (1.00-1.12) | LR | 1.12 (1.00-1.25) | 1.16  (1.04-1.29) |
| Persons | 1.87 (1.61-2.17) | 1.25 (1.10-1.42) | 1.18 (1.06-1.31) | 1.21 (1.07-1.38) | 1.10 (1.06-1.13) | 1.28 (1.24-1.33) | LR | LR | LR | 1.40 (1.18-1.66) | 1.10 (1.00-1.23) | LR | 1.08 (1.00-1.31) | 1.34  (1.20-1.48) | 1.04 (1.00-1.15) | LR | LR | 1.06 (1.00-1.12) | LR | 1.09 (1.04-1.14) | 1.12  (1.07-1.18) |
| ***Obese vs healthy weight (BMI 30+ vs BMI 18.5<25)*** | | | | | | | | | | | | | | | | | | | | |  |
| Male | 2.73 (2.16-3.46) | 1.48 (1.15-1.92) | 1.83 (1.59-2.11) | 1.33 (1.25-1.42) | NA | 1.59 (1.38-1.83) | LR | LR | LR | 1.93 (1.52-2.45) | 1.54 (1.25-1.89) | LR | NA | NA | 1.14 (1.00-1.30) | NA | LR | 1.19 (1.04-1.37) | 1.36 (1.09-1.69) | 1.40 (1.24-1.57) | 1.17  (1.01-1.34) |
| Female | 2.73 (2.16-3.46) | 1.48 (1.15-1.92) | 1.83 (1.59-2.11) | 1.33 (1.25-1.42) | 1.18 (1.12-1.25) | 1.91 (1.75-2.08) | LR | LR | LR | 1.93 (1.52-2.45) | 1.75 (1.44-2.14) | LR | 1.27 (1.19-1.35) | 2.54  (2.27-2.81) | NA | LR | LR | 1.19 (1.04-1.37) | 1.61 (1.34-1.93) | 1.20 (1.07-1.35) | 1.27  (1.15-1.40) |
| Persons | 2.73 (2.16-3.46) | 1.48 (1.15-1.92) | 1.83 (1.59-2.11) | 1.33 (1.25-1.42) | 1.18 (1.12-1.25) | 1.77 (1.68-1.87) | LR | LR | LR | 1.93 (1.52-2.45) | 1.58 (1.43-1.75) | LR | 1.27 (1.19-1.35) | 2.54  (2.27-2.81) | 1.14 (1.00-1.30) | LR | LR | 1.19 (1.04-1.37) | 1.20 (1.07-1.35) | 1.26 (1.17-1.37) | 1.21  (1.08-1.35) |
|  |  |  | | | | | | | | | | | | | | | | | | |  |
| ***Alcohol consumption***[22–29] | | | | | | | | | | | | | | | | | | | | |  |
| ***Current drinker Vs no/occasional drinker*** | | | | | | | | | | | | | | | | | | | | |  |
| Male | LR | LR | 1.14  (1-1.34) | 1.25 (1.13-1.39) | NA | LR | 1.17 (1.01-1.35) | 1.10 (1.01-1.19) | 1.62 (1.27-2.08) | 1.21 (10.7-1.36) | LR | LR | NA | LR | LR | NA | LR | LR | LR | LR | LR |
| Female | LR | LR | 1.45 (1.01-1.90) | 1.00 (1.00-1.07) | 1.11 (1.06-1.17) | LR | 1.17 (1.01-1.35) | 1.10 (1.01-1.19) | 1.62 (1.27-2.08) | 1.21 (10.7-1.36) | LR | LR | LR | LR | NA | LR | LR | LR | LR | LR | LR |
| Persons | LR | LR | 1.28 (1.13-1.44) | 1.12 (1.06-1.19) | 1.11 (1.06-1.17) | LR | 1.17 (1.01-1.35) | 1.10 (1.01-1.19) | 1.62 (1.27-2.08) | 1.21 (10.7-1.36) | LR | LR | LR | LR | LR | LR | LR | LR | LR | LR | LR |
|  |  |  | | | | | | | | | | | | | | | | | | |  |
| ***Physical activity* (*600-3999 vs <600 MET-minutes per week)***[30, 31] | | | | | | | | | | | | | | | | | | | | |  |
| Male | LR | LR | LR | 0.90 (0.85-0.95) | NA | LR | LR | LR | LR | LR | LR | LR | NA | LR | LR | NA | LR | LR | LR | LR | LR |
| Female | LR | LR | LR | 0.90 (0.85-0.95) | 0.96  (0.93-0.99) | LR | LR | LR | LR | LR | LR | LR | LR | LR | NA | LR | LR | LR | LR | LR | LR |
| Persons | LR | LR | LR | 0.90 (0.85-0.95) | 0.96  (0.93-0.99) | LR | LR | LR | LR | LR | LR | LR | LR | LR | LR | LR | LR | LR | LR | LR | LR |
|  |  |  | | | | | | | | | | | | | | | | | | |  |
| ***Tobacco (cigarrete) smoking***[32–39] | | | | | | | | | | | | | | | | | | | | |  |
| ***Current smoker vs never*** | | | | | | | | | | | | | | | | | | | | |  |
| Male | 2.50 (2.00-3.13) | 1.63 (1.32-2.03) | 1.85 (1.21-2.83) | 1.38 (1.22-1.56) | NA | 1.59 (1.32-1.91) | 3.43 (2.37-4.94) | 1.95 (1.31-2.91) | 8.07 (3.94-16.54) | 1.74 (1.46-2.07) | LR | 2.81  (2.27-3.47) | NA | LR | LR | NA | 9.87 (6.85-14.24) | LR | LR | 1.42 (1.12-1.81) | LR |
| Female | 2.50 (2.00-3.13) | 1.73 (1.31-2.30) | 1.49 (1.12-1.98) | 1.06 (0.95-1.19) | LR | 1.35 (1.05-1.73) | 3.43 (2.37-4.94) | 1.95 (1.31-2.91) | 8.07 (3.94-16.54) | 1.45 (1.20-1.75) | LR | 2.81  (2.27-3.47) | 1.49 (1.17-1.89) | LR | NA | 1.83 (1.51-2.21) | 7.58 (5.36-10.73) | LR | LR | 1.28 (1.03-1.60) | LR |
| Persons | 2.50 (2.00-3.13) | 1.70 (1.51-1.91) | 1.56 (1.29-1.87) | 1.20 (1.10-1.30) | LR | 1.52 (1.33-1.74) | 3.43 (2.37-4.94) | 1.95 (1.31-2.91) | 8.07 (3.94-16.54) | 1.64 (1.37-1.95) | LR | 2.81  (2.27-3.47) | 1.49 (1.17-1.89) | LR | LR | 1.83 (1.51-2.21) | 8.96 (6.73-12.10) | LR | LR | 1.46 (1.15-1.86) | LR |
|  |  |  | | | | | | | | | | | | | | | |  |  |  |  |
| ***Former smoker vs never*** | | | | | | | | | | | | | | | | | | | | |  |
| Male | 2.03 (1.77-2.33) | 1.18 (1.04-1.33) | 1.49 (1.06-2.10) | 1.23 (1.09-1.40) | NA | 1.25 (1.14-1.37) | 1.40 (1.00-2.00) | 1.39 (1.08-1.79) | 2.63 (1.26-5.47) | 1.31 (1.17-1.46) | LR | 1.92  (1.72-2.15) | NA | LR | LR | NA | 3.85 (2.77-5.34) | LR | LR | LR | LR |
| Female | 2.03 (1.77-2.33) | 1.18 (1.04-1.33) | 1.49 (1.06-2.10) | 1.18 (1.08-1.28) | LR | 1.25 (1.14-1.37) | 1.40 (1.00-2.00) | 1.39 (1.08-1.79) | 2.63 (1.26-5.47) | 1.31 (1.17-1.46) | LR | 1.92  (1.72-2.15) | 1.08 (1.00-1.33) | LR | NA | 1.26 (1.11-1.42) | 3.85 (2.77-5.34) | LR | LR | LR | LR |
| Persons | 2.03 (1.77-2.33) | 1.18 (1.04-1.33) | 1.49 (1.06-2.10) | 1.18 (1.12-1.25) | LR | 1.25 (1.14-1.37) | 1.40 (1.00-2.00) | 1.39 (1.08-1.79) | 2.63 (1.26-5.47) | 1.31 (1.17-1.46) | LR | 1.92  (1.72-2.15) | 1.08 (1.00-1.33) | LR | LR | 1.26 (1.11-1.42) | 3.85 (2.77-5.34) | LR | LR | LR | LR |
| *^a^ Relative risks obtained only for cancer type-risk factor combinations classified as sufficient/convincing by IARC and WCRF.*  *RR = 1 if cancer type-risk factor association is not significant from the chosen source.*  *If there is no specific RR stratified for genders, overall RR will be used for calculation of PAF.* | | | | | | | | | | | | | | | | | | | | |  |
| *NA=Not applicable; LR=Limited risk* | | | | | | | | | | | | | | | | | | | | |  |

**Table S4** Summary of PAFs of each modifiable risk factors all cancer sites

| **Cancers** | **Overweight and Obesity** | | | **Alcohol Consumption** | | | **Physical Inactivity** | | | **Tobacco Smoking (current and former smoking)** | | |
| --- | --- | --- | --- | --- | --- | --- | --- | --- | --- | --- | --- | --- |
|  | Male | Female | Persons | Male | Female | Persons | Male | Female | Persons | Male | Female | Persons |
| Oesophageal | 30.1  (22.3-38.1) | 35.5  (26.7-44.0) | 33.1  (24.9-41.3) | - | - | - | - | - | - | 44.7  (34.9-53.8) | 3.2  (1.9-4.9) | 27.4  (20.0-35.2) |
| Pancreas | 10.9  (4.1-18.4) | 13.4  (5.0-22.4) | 12.3  (4.7-20.5) | - | - | - | - | - | - | 23.8  (13.0-34.5) | 1.3  (0.5-2.6) | 13.8  (9.8-17.9) |
| Liver | 12.0  (6.8-17.4) | 16.4  (10.3-22.5) | 14.4  (8.9-20.2) | 1.9  (0-4.8) | 1.8  (0-3.9) | 2.3  (1.0-3.9) | - | - | - | 31.0  (9.2-49.9) | 1.2  (0.2-2.8) | 12.8  (6.0-20.2) |
| Colorectal | 8.7  (4.2-13.8) | 10.5  (5.8-15.8) | 9.7  (5.1-14.8) | 3.3  (1.6-5.4) | 0  (0-0.3) | 1.0  (0.5-1.7) | 3.5  (1.7-5.5) | 4.8  (2.4-7.6) | 4.2  (2.1-6.7) | 16.8  (9.9-23.8) | 0.3  (0.1-0.6) | 5.0  (2.6-7.4) |
| Breast | NA | 5.7  (3.6-7.7) | 5.7  (3.6-7.7) | NA | 0.4  (0.2-0.8) | 0.4  (0.2-0.8) | NA | 1.7  (0.1-3.2) | 1.7  (0.1-3.2) | NA | - | - |
| Endometrial | NA | 26.8  (21.1-31.8) | 26.8  (21.1-31.8) | - | - | - | - | - | - | - | - | - |
| Kidney | 11.1  (5.9-16.5) | 22.8  (17.5-28.1) | 15.9  (13.9-18.3) | - | - | - | - | - | - | 23.2  (13.8-32.2) | 0.8  (0.2-1.7) | 11.1  (7.1-15.5) |
| Oral cavity | - | - | - | 2.3  (0.1-4.9) | 0.7  (0-1.6) | 1.4  (0.1-3.1) | - | - | - | 54.0  (38.4-66.5) | 4.1  (1.9-7.5) | 35.2  (22.3-48.0) |
| Nasopharynx | - | - | -- | 1.4  (0.1-2.7) | 0.4  (0-0.9) | 0.8  (0.1-1.7) | - | - | - | 32.6  (13.0-49.9) | 1.8  (0.5-4.1) | 18.4  (6.5-31.7) |
| Larynx | - | - | - | 7.8  (3.3-13.7) | 2.4  (0.9-4.6) | 5.0  (2.1-9.0) | - | - | - | 77.6  (57.7-88.7) | 11.3  (4.1-24.5) | 61.7  (38.7-78.6) |
| Stomach | 17.5  (9.2-26.1) | 21.6  (12.1-31.2) | 19.8  (10.9-28.9) | 2.8  (0.9-5.0) | 0.8  (0.2-1.6) | 1.8  (0.5-3.2) | - | - | - | 27.4  (18.5-35.6) | 1.0  (0.4-1.8) | 13.4  (8.0-19.0) |
| Gallbladder | 5.4  (2.3-11.5) | 16.2  (9.0-23.9) | 9.9  (5.5-15.0) | - | - | - | - | - | - | - | - | - |
| Bladder | - | - | - | - | - | - | - | - | - | 48.5  (39.5-56.6) | 3.6  (2.2-5.3) | 30.5  (23.3-37.8) |
| Ovary | NA | 6.5  (3.1-13.3) | 6.5  (3.1-13.3) | NA | - | - | NA | - | - | NA | 0.9  (0.2-1.9) | 0.9  (0.2-1.9) |
| Prostate | 2.5  (0-7.2) | NA | 2.5  (0-7.2) | - | NA | - | - | NA | - | - | NA | - |
| Cervix | NA | - | - | NA | - | - | NA | - | - | NA | 1.5  (0.8-2.5) | 1.5  (0.8-2.5) |
| Lung | - | - | - | - | - | - | - | - | - | 81.6  (74.0-87.1) | 11.6  (6.8-17.9) | 65.1  (56.4-72.9) |
| Non-Hodgkin’s Lymphoma | 3.6  (0.4-7.0) | 4.8  (0.7-9.2) | 4.2  (0.5-8.2) | - | - | - | - | - | - | - | - | - |
| Hodgkin’s Lymphoma | 3.5  (0.9-6.8) | 9.6  (5.4-14.3) | 5.4  (1.9-9.8) | - | - | - | - | - | - | - | - | - |
| Leukaemia | 5.2  (2.2-9.1) | 6.5  (1.2-12) | 5.9  (3.3-8.7) | - | - | - | - | - | - | 16.3  (5.2-27.7) | 0.5  (0.04-1.07) | 9.0  (3.1-15.9) |
| Multiple Myeloma | 5.0  (1.8-8.6) | 8.5  (3.5-13.6) | 6.0  (3.0-9.4) | - | - | - | - | - | - | - | - | - |

**Table S5** Attributable cases of each modifiable risk factors for related cancer types

|  | **Overweight and Obesity** | | | **Alcohol Consumption** | | | **Physical Inactivity** | | | **Tobacco Smoking (current and former smoking)** | | |
| --- | --- | --- | --- | --- | --- | --- | --- | --- | --- | --- | --- | --- |
|  | Male | Female | Persons | Male | Female | Persons | Male | Female | Persons | Male | Female | Persons |
| Oesophageal | 90  (66-113) | 44  (33-55) | 140  (105-174) | - | - | - | - | - | - | 133  (104-160) | 4  (2-6) | 116  (84-149) |
| Pancreas | 59  (22-99) | 59  (22-98) | 120  (46-200) | - | - | - | - | - | - | 128  (70-186) | 6  (2-11) | 135  (96-175) |
| Liver | 175  (100-254) | 79  (50-109) | 280  (173-393) | 27  (0-69) | 9  (0-19) | 45  (20-75) | - | - | - | 452  (135-728) | 6  (1-13) | 249  (117-393) |
| Colorectal | 291  (141-461) | 294  (161-440) | 595  (315-909) | 111  (54-181) | 0  (0-9) | 62  (29-104) | 116  (56-183) | 134  (68-213) | 257  (129-408) | 561  (330-794) | 7  (0-17) | 307  (162-455) |
| Breast | NA | 429  (269-583) | 429  (269-583) | NA | 33  (16-58) | 33  (16-58) | NA | 128  (8-246) | 128  (8-246) | NA | - | - |
| Endometrial | NA | 294  (232-350) | 294  (232-350) | - | - | - | - | - | - | - | - | - |
| Kidney | 62  (33-93) | 74  (56-91) | 141  (123-162) | - | - | - | - | - | - | 131  (78-181) | 3  (1-5) | 99  (63-138) |
| Oral cavity | - | - | - | 8  (0-16) | 2  (0-5) | 10  (1-21) | - | - | - | 181  (129-223) | 14  (6-25) | 235  (149-320) |
| Nasopharynx | - | - | -- | 22  (2-43) | 2  (0-4) | 18  (2-36) | - | - | - | 522  (208-798) | 9  (2-20) | 384  (135-663) |
| Larynx | - | - | - | 34  (14-59) | 1  (0-2) | 24  (10-42) | - | - | - | 335  (249-383) | 4  (2-9) | 290  (182-369) |
| Stomach | 152  (80-227) | 161  (90-233) | 319  (176-466) | 24  (8-44) | 6  (2-12) | 28  (9-51) | - | - | - | 238  (161-312) | 7  (3-13) | 216  (128-307) |
| Gallbladder | 8  (3-17) | 33  (18-48) | 35  (19-52) | - | - | - | - | - | - | - | - | - |
| Bladder | - | - | - | - | - | - | - | - | - | 303  (247-354) | 7  (4-10) | 250  (191-310) |
| Ovary | NA | 83  (39-169) | 83  (39-169) | NA | - | - | NA | - | - | NA | 11  (3-24) | 11  (3-24) |
| Prostate | 46  (0-130) | NA | 46  (0-130) | - | NA | - | - | NA | - | - | NA | - |
| Cervix | NA | - | - | NA | - | - | NA | - | - | NA | 26  (13-43) | 26  (13-43) |
| Lung | - | - | - | - | - | - | - | - | - | 2795  (2535-2985) | 146  (86-226) | 3051  (2643-3414) |
| Non-Hodgkin’s Lymphoma | 36  (4-72) | 37  (5-72) | 76  (10-147) | - | - | - | - | - | - | - | - | - |
| Hodgkin’s Lymphoma | 5  (1-9) | 7  (4-10) | 11  (4-20) | - | - | - | - | - | - | - | - | - |
| Leukaemia | 49  (21-86) | 50  (9-92) | 101  (57-149) | - | - | - | - | - | - | 155  (49-263) | 3  (0-8) | 155  (52-273) |
| Multiple Myeloma | 6  (2-11) | 8  (3-14) | 14  (7-21) | - | - | - | - | - | - | - | - | - |

**Table S6 Summary of combined PAF (%) based on cancer sites in Malaysia in 2018**

| **Types of Cancer** | **Male** | | **Female** | | **Persons** | |
| --- | --- | --- | --- | --- | --- | --- |
|  | **Combined PAF**  **% (95%CI)** | **Attributable cases,**  **n (95% CI)** | **Combined PAF**  **% (95%CI)** | **Attributable cases,**  **n (95% CI)** | **Combined PAF**  **% (95%CI)** | **Attributable cases,**  **n (95% CI)** |
|  |  |  |  |  |  |  |
| Oesophageal | 61.4  (49.4-71.4) | 183  (147-213) | 37.6  (28.1-46.7) | 47  (35-58) | 51.5  (39.9-62.0) | 217  (168-262) |
| Pancreas | 32.1  (16.6-46.5) | 173  (90-251) | 14.6  (5.5-24.4) | 64  (24-107) | 24.4  (14.1-34.8) | 238  (137-339) |
| Liver | 40.4  (15.4-60.6) | 590  (225-884) | 18.9  (10.6-27.6) | 91  (51-134) | 27.1  (15.2-38.8) | 527  (296-753) |
| Colorectal | 29.1  (16.5-41.2) | 972  (551-1378) | 15.0  (8.0-22.9) | 420  (225-640) | 18.5  (10.0-27.6) | 1144  (613-1694) |
| Breast | NA | NA | 7.7  (3.8-11.3) | 581  (292-861) | 7.7  (3.8-11.3) | 581  (292-861) |
| Endometrial | NA | NA | 26.8  (21.1-31.8) | 294  (232-350) | 26.8  (21.1-31.8) | 294  (232-350) |
| Kidney | 31.7  (18.8-43.3) | 179  (106-244) | 23.4  (17.6-29.3) | 76  (57-95) | 25.3  (20.0-31.0) | 224  (177-275) |
| Oral cavity | 55.0  (38.5-68.1) | 184  (129-228) | 4.7  (1.9-8.9) | 16  (6-30) | 36.2  (22.4-49.6) | 241  (149-331) |
| Nasopharynx | 33.5  (13.1-51.3) | 536  (210-820) | 2.2  (0.5-4.9) | 11  (3-24) | 19.1  (6.5-32.9) | 398  (137-687) |
| Larynx | 79.3  (59.1-90.3) | 343  (255-390) | 13.5  (5.0-28.0) | 5  (2-11) | 63.6  (39.9-80.5) | 299  (188-378) |
| Stomach | 41.8  (26.7-55.0) | 363  (231-478) | 23.1  (12.6-33.5) | 172  (94-250) | 31.7  (18.4-44.2) | 512  (297-714) |
| Gallbladder | 5.4  (2.3-11.5) | 8  (3-17) | 16.2  (9.0-23.9) | 33  (18-48) | 9.9  (5.5-15.0) | 35  (19-52) |
| Bladder | 48.5  (39.5-56.6) | 303  (247-354) | 3.6  (2.2-5.3) | 7  (4-10) | 30.5  (23.3-37.8) | 250  (191-310) |
| Ovary | NA | NA | 7.3  (3.3-15.0) | 93  (42-190) | 7.3  (3.3-15.0) | 93  (42-190) |
| Prostate | 2.5  (0-7.2) | 46  (0-130) | NA | NA | 2.5  (0-7.2) | 46  (0-130) |
| Cervix | NA | NA | 1.5  (0.8-2.5) | 26  (13-43) | 1.5  (0.8-2.5) | 26  (13-43) |
| Lung | 81.6  (74.0-87.1) | 2795  (2535-2985) | 11.6  (6.8-17.9) | 146  (86-226) | 65.1  (56.4-72.9) | 3051  (2643-3414) |
| Non-Hodgkin’s Lymphoma | 3.6  (0.4-7.0) | 36  (4-72) | 4.8  (0.7-9.2) | 37  (5-72) | 4.2  (0.5-8.2) | 76  (10-147) |
| Hodgkin’s Lymphoma | 3.5  (0.8-6.8) | 5  (1-9) | 9.6  (5.4-14.3) | 7  (4-10) | 5.4  (1.9-9.8) | 11  (4-20) |
| Leukaemia | 20.7  (7.3-34.3) | 196 (69-325) | 6.9  (1.2-12.9) | 53  (9-99) | 14.4  (6.3-23.2) | 247  (108-399) |
| Multiple Myeloma | 5.0  (1.8-8.6) | 6  (2-11) | 8.5  (3.5-13.6) | 8  (3-14) | 6.0  (3.0-9.4) | 14  (7-21) |
| **Total** | **39.1**  **(27.2-49.7)** | **6917**  **(4807-8789)** | **10.5**  **(5.8-15.7)** | **2186**  **(1206-3270)** | **22.2**  **(14.9-29.6)** | **8523**  **(5724-11371)** |

**References**

1. Dr Ahmad Faudzi, Dr Mohd Azahadi Omar, Dr Nik Daliana Nik Farid, Mohd Hanafi bin Abdullah, Dr Jamalludin Ab Rahman MKC. The Third National Health and Morbidity Survey 2006 (NHMS III). 2008.

2. Ferlay J, Ervik M, Lam F et al. Global Cancer Observatory (GCO): Cancer today.

3. Turati F, Tramacere I, La Vecchia C, Negri E. A meta-analysis of body mass index and esophageal and gastric cardia adenocarcinoma. Ann Oncol. 2013;24:609–17. doi:10.1093/annonc/mds244.

4. Munsell MF, Sprague BL, Berry DA, Chisholm G, Trentham-Dietz A. Body mass index and breast cancer risk according to postmenopausal estrogen-progestin use and hormone receptor status. Epidemiol Rev. 2014;36:114–36.

5. Alsamarrai A, Das SLM, Windsor JA, Petrov MS. Factors That Affect Risk for Pancreatic Disease in the General Population: A Systematic Review and Meta-analysis of Prospective Cohort Studies. Clin Gastroenterol Hepatol. 2014;12:1635-1644.e5. doi:10.1016/j.cgh.2014.01.038.

6. Harrison S, Tilling K, Turner EL, Martin RM, Lennon R, Lane JA, et al. Systematic review and meta-analysis of the associations between body mass index, prostate cancer, advanced prostate cancer, and prostate-specific antigen. Cancer Causes Control. 2020;31:431–49. doi:10.1007/s10552-020-01291-3.

7. Castillo JJ, Reagan JL, Ingham RR, Furman M, Dalia S, Merhi B, et al. Obesity but not overweight increases the incidence and mortality of leukemia in adults: A meta-analysis of prospective cohort studies. Leuk Res. 2012;36:868–75.

8. Psaltopoulou T, Sergentanis TN, Ntanasis-Stathopoulos I, Tzanninis IG, Riza E, Dimopoulos MA. Anthropometric characteristics, physical activity and risk of hematological malignancies: A systematic review and meta-analysis of cohort studies. Int J Cancer. 2019;145:347–59.

9. Willett E V, Morton LM, Hartge P, Becker N, Boffetta P, Bracci P, et al. InterLymph consortium. 2014;122:2062–70.

10. Larsson SC, Wolk A. Body mass index and risk of non-Hodgkin’s and Hodgkin’s lymphoma: A meta-analysis of prospective studies. Eur J Cancer. 2011;47:2422–30. doi:10.1016/j.ejca.2011.06.029.

11. Larsson SC, Wolk A. Obesity and risk of non-Hodgkin’s lymphoma: A meta-analysis. Int J Cancer. 2007;121:1564–70.

12. Jenabi E, Poorolajal J. The effect of body mass index on endometrial cancer : a meta-analysis. Public Health. 2015;129:872–80. doi:10.1016/j.puhe.2015.04.017.

13. Wallin A, Larsson SC. Body mass index and risk of multiple myeloma : A meta-analysis of prospective studies 5. Eur J Cancer. 2011;47:1606–15. doi:10.1016/j.ejca.2011.01.020.

14. Okabayashi K, Ashrafian H, Hasegawa H, Yoo JH, Patel VM, Harling L, et al. Body mass index category as a risk factor for colorectal adenomas: A systematic review and meta-analysis. Am J Gastroenterol. 2012;107:1175–85.

15. Li ZM, Wu ZX, Han B, Mao YQ, Chen HL, Han SF, et al. The association between BMI and gallbladder cancer risk: A meta-analysis. Oncotarget. 2016;7:43669–79.

16. Wang F, Xu Y. Body mass index and risk of renal cell cancer: A dose-response meta-analysis of published cohort studies. Int J Cancer. 2014;135:1673–86.

17. Chen Y, Wang X, Wang J, Yan Z, Luo J. Excess body weight and the risk of primary liver cancer: An updated meta-analysis of prospective studies. Eur J Cancer. 2012;48:2137–45. doi:10.1016/j.ejca.2012.02.063.

18. Xue K, Li FF, Chen YW, Zhou YH, He J. Body mass index and the risk of cancer in women compared with men: A meta-analysis of prospective cohort studies. Eur J Cancer Prev. 2017;26:94–105.

19. Ma Y, Yang Y, Wang F, Zhang P, Shi C, Zou Y, et al. Obesity and Risk of Colorectal Cancer: A Systematic Review of Prospective Studies. PLoS One. 2013;8.

20. Poorolajal J, Jenabi E, Masoumi SZ. Body mass index effects on risk of Ovarian cancer: A meta-analysis. Asian Pacific J Cancer Prev. 2014;15:7665–71.

21. Aune D, Greenwood DC, Chan DSM, Vieira R, Vieira AR, Navarro Rosenblatt DA, et al. Body mass index, abdominal fatness and pancreatic cancer risk: A systematic review and non-linear dose-response meta-analysis of prospective studies. Ann Oncol. 2012;23:843–52. doi:10.1093/annonc/mdr398.

22. Key J, Hodgson S, Omar RZ, Jensen TK, Thompson SG, Boobis AR, et al. Meta-analysis of studies of alcohol and breast cancer with consideration of the methodological issues. Cancer Causes Control. 2006;17:759–70.

23. Fedirko V, Tramacere I, Bagnardi V, Rota M, Scotti L, Islami F, et al. Alcohol drinking and colorectal cancer risk: An overall and dose-Response meta-analysis of published studies. Ann Oncol. 2011;22:1958–72.

24. Ferro A, Morais S, Rota M, Pelucchi C, Bertuccio P, Bonzi R, et al. Alcohol intake and gastric cancer: Meta-analyses of published data versus individual participant data pooled analyses (StoP Project). Cancer Epidemiol. 2018;54 April:125–32.

25. Islami F, Tramacere I, Rota M, Bagnardi V, Fedirko V, Scotti L, et al. Alcohol drinking and laryngeal cancer: Overall and dose-risk relation - A systematic review and meta-analysis. Oral Oncol. 2010;46:802–10. doi:10.1016/j.oraloncology.2010.07.015.

26. Chuang SC, Lee YCA, Wu GJ, Straif K, Hashibe M. Alcohol consumption and liver cancer risk: a meta-analysis. Cancer Causes Control. 2015;26:1205–31.

27. Li Y, Yang H, Cao J. Association between alcohol consumption and cancers in the chinese population-a systematic review and meta-analysis. PLoS One. 2011;6.

28. Du T, Chen K, Zheng S, Bao M, Huang Y, Wu K. Association Between Alcohol Consumption and Risk of Nasopharyngeal Carcinoma: A Comprehensive Meta-Analysis of Epidemiological Studies. Alcohol Clin Exp Res. 2019;43:2262–73.

29. Turati F, Garavello W, Tramacere I, Bagnardi V, Rota M, Scotti L, et al. A meta-analysis of alcohol drinking and oral and pharyngeal cancers. Part 2: Results by subsites. Oral Oncol. 2010;46:720–6. doi:10.1016/j.oraloncology.2010.07.010.

30. Kyu HH, Bachman VF, Alexander LT, Mumford JE, Afshin A, Estep K, et al. Physical activity and risk of breast cancer, colon cancer, diabetes, ischemic heart disease, and ischemic stroke events: Systematic review and dose-response meta-analysis for the Global Burden of Disease Study 2013. BMJ. 2016;354:1–10.

31. Wu Y, Zhang D, Kang S. Physical activity and risk of breast cancer: A meta-analysis of prospective studies. Breast Cancer Res Treat. 2013;137:869–82.

32. Gandini S, Botteri E, Iodice S, Boniol M, Lowenfels AB, Maisonneuve P, et al. Tobacco smoking and cancer: A meta-analysis. Int J Cancer. 2008;122:155–64.

33. Tsoi KKF, Pau CYY, Wu WKK, Chan FKL, Griffiths S, Sung JJY. Cigarette Smoking and the Risk of Colorectal Cancer: A Meta-analysis of Prospective Cohort Studies. Clin Gastroenterol Hepatol. 2009;7:682-688.e5. doi:10.1016/j.cgh.2009.02.016.

34. Maasland DHE, van den Brandt PA, Kremer B, Goldbohm RA(., Schouten LJ. Alcohol consumption, cigarette smoking and the risk of subtypes of head-neck cancer: Results from the Netherlands Cohort Study. BMC Cancer. 2014;14:1–14. doi:10.1186/1471-2407-14-187.

35. Pandeya N, Williams GM, Sadhegi S, Green AC, Webb PM, Whiteman DC. Associations of duration, intensity, and quantity of smoking with adenocarcinoma and squamous cell carcinoma of the esophagus. Am J Epidemiol. 2008;168:105–14.

36. Calle EE, Gapstur SM, Patel A V., Dal Maso L, Talamini R, Chetrit A, et al. Ovarian cancer and smoking: Individual participant meta-analysis including 28 114 women with ovarian cancer from 51 epidemiological studies. Lancet Oncol. 2012;13:946–56. doi:10.1016/S1470-2045(12)70322-4.

37. Fircanis S, Merriam P, Khan N, Castillo JJ. The relation between cigarette smoking and risk of acute myeloid leukemia: An updated meta-analysis of epidemiological studies. Am J Hematol. 2014;89:125–32.

38. Shi H, Shao X, Hong Y. Association between cigarette smoking and the susceptibility of acute myeloid leukemia: A systematic review and meta-analysis. Eur Rev Med Pharmacol Sci. 2019;23:10049–57.

39. Cumberbatch MG, Rota M, Catto JWF, La C, Catto J. The Role of Tobacco Smoke in Bladder and Kidney Carcinogenesis : A Comparison of Exposures and Meta-analysis of Incidence and Mortality Risks. Eur Urol. 2016;70:458–66. doi:10.1016/j.eururo.2015.06.042.
